# Identifying the vulnerable regions of emergency medical services based on the three-stage of accessibility: a case study in Xi’an, China

**DOI:** 10.1186/s12939-022-01653-0

**Published:** 2022-04-22

**Authors:** Ning Xu, Jianjun Bai, Ran Yan

**Affiliations:** School of Geography and Tourism, Shaanxi Normal University, Xi’an, 710119 China

**Keywords:** Three stages, Accessibility of emergency care, Identifying vulnerable regions, Xi’an, China

## Abstract

**Background:**

Emergency department crowding is an obstacle in the process of obtaining emergency care services, which will lead to the increase of time cost. Most studies focused on the direct access to emergency medical resources, and few studies took the crowding of hospital emergency department as an evaluation index to reflect the convenience of obtaining emergency medical resources. It is a significance for the identification of areas with insufficient access to emergency service resources with this method.

**Methods:**

This paper utilizes the improved potential model and the inverted Two-Step Floating Catchment Area method, combined with network map API service data to evaluate response time, delivery time and waiting time (for emergency department crowding) spent in different residential areas of Xi’an City in the process of emergency. Meanwhile, the vulnerable regions of gaining emergency medical resources are identified through the comprehensive analysis of the three stages of emergency.

**Results:**

The studies show that the residents in built-up area are more convenient to get ambulance service and arrive at care hospitals than those in suburban areas, but they may face greater hospital crowdedness. Although suburban residents are faced with low hospital crowdedness, they spend more time on getting ambulances and going to care hospitals. The accessibility of emergency medical resources varies greatly among residents in different regions, with 5.38% of the residents were identified in the high-risk area distributing in suburban residential areas in the south of the city center, 21.92% in the medium risk area in the southern mountainous areas and the periphery of the core suburban areas of the city, and 46.11% in the low-risk area which are mainly distributed in built-up areas in gaining emergency medical services.

**Conclusions:**

Obviously, getting an ambulance and arriving at the nearest hospital quickly shows that it is conducive to access to emergency resources. However, the impact of hospital emergency crowding can not be ignored, especially in the area surrounded by high-grade hospitals in the central area of the city. In considering the spatial layout of emergency stations and emergency hospitals, the dislocation distribution of hospitals at different levels should be reasonably adjusted to balance the equity of residents in obtaining emergency medical resources.

## Background

Emergency medical service (EMS) is a service network that provides emergency medical resources, including caring at the rescue site, caring on the way to hospital, as well as receiving and providing immediate care to patients in hospital [1]. As a kind of basic public service resource, it bears the heavy burden of urban emergency relief and residents’ life safety [2]. Therefore, a good emergency service system plays an important role in improving the life security and satisfaction of urban residents [3].

Response, ambulance delivery and hospital care are included in the EMS system, and the quality of these variables determines the service level of the system [4–7]. The results of EMS system evaluation can provide reference for the development of emergency medicine and perfect medical facilities [8]. The United States and some European countries have adopted some standards to formulate relevant evaluation systems to discuss the stability (quality) of the emergency system. Time is a key factor, including the response time from receiving the emergency call to the team arriving at the place where the incident occurred, and the time transporting the patient to the appropriate care institutions [9]. In addition, when ambulances transport patients to medical institutions, they may face the situation of crowdedness, and nurses need to provide patients care until they have emergency beds [10]. This delay is called ambulance unloading delay (AOD), which is usually caused by over-crowdedness in medical institutions [11, 12]. Long response delay time of the emergency system may lead to the reduced survival rate of the patients [13]. Similarly, it is vital to transport the patients to the medical institutions by ambulances in a prime time to save lives [14]. In addition to the above two phases, AOD is also an indispensable factor in evaluating the level of the whole emergency system [15]. In light of the above insights, we can find that there are three critical stages from the initial emergency call to placing the patient in a hospital bed, including the response of ambulance, the delivery and unloading of patients. Time is very important in this process, and it is feasible to evaluate the level of emergency services using the time cost in each stage. While the vulnerable areas for emergency services can be identified by the time cost [16, 17].

The earliest evaluation of rescue geographic accessibility focused on the number of rescue units, without considering the location and time [18]. However, time is the key factor in the process of emergency, and risks in some areas can be identified according to the “golden time” by using accurate travel time [19, 20]. Then, with the development of geographic information technology, geographic information system (GIS) was gradually used to evaluate the time in emergency system [21]. In the study of response time, the accessibility method that used road network data was also applied to evaluate the time distance of ambulance transportation [22]. The travel time is mostly calculated by using some standard road speeds with network analysis, but the application of this method in the evaluation of emergency obviously cannot reflect the actual situation of road traffic [23]. There are also some scholars directly evaluated the response time by recorded measured data and GIS simulation [24], while Jaldell determined the monetary value of response time with the marginal effect, and evaluated the response time of emergency by using the value of death or serious injury per minute, which can better reflect the importance of response time, and also well prove the truth that “time is money” [25]. However, this is an evaluation under the ideal state of no supply and no competitive pressure. Actually, there are problems in the acquisition or allocation of resources, such as whether the supply is sufficient and whether the demand is satisfied. Therefore, on the basis of time, some scholars considered the impact of population distribution and service capacity of facilities on the accessibility of emergency care [26–28]. The method of accessibility not only can well reflect the difficulty of obtaining emergency resources, also give full play to the advantages of geographical distribution to optimize the allocation of resources. In terms of delivery time, Adam Roberts et al. used medical statistics to explore how changes in the geographical distribution of emergency care institutions affect the average distance from home to hospital, and found that the time to go to the care institution increased with the increase of distance, which could increase the risk of death in the process to get further treatment [29]. In addition, related research on waiting time, some scholars replaced the waiting time problem caused by ambulance “unloading delay” with hospitalization time or turnover interval, and got better evaluation results. Studies have shown that longer average hospitalization time or slower turnover efficiency may lead to longer waiting time [30]. These studies measured the real time of emergency by evaluating alternative variables from different perspectives, and illustrated that excessive cost in any period of time during the emergency could cause certain risks in ensuring life safety. However, relevant studies did not pay attention to the whole process of emergency and focus only on one stage of the EMS, so that cannot comprehensively and accurately evaluate the convenience of access to emergency medical care in different areas, and cannot well identify the vulnerable areas of access to emergency medical resources.

In this research, Xi’an City, which has a relatively complete emergency system, is selected as the research area. The time spent in the emergency process is divided into three stages, including response time, delivery time and waiting time for ambulance unloading delay. The ambulance accessibility value, the shortest distance time and the hospital crowdedness are respectively used to evaluate the above three stage time. And the Amap API data (https://lbs.amap.com/) is used in the travel time involved to get a more real situation. The results of each stage are used to systematically evaluate the level of emergency system, and identify the vulnerable areas of emergency services with the comprehensive analysis.

## Materials

### Study area

**Fig. 1:**
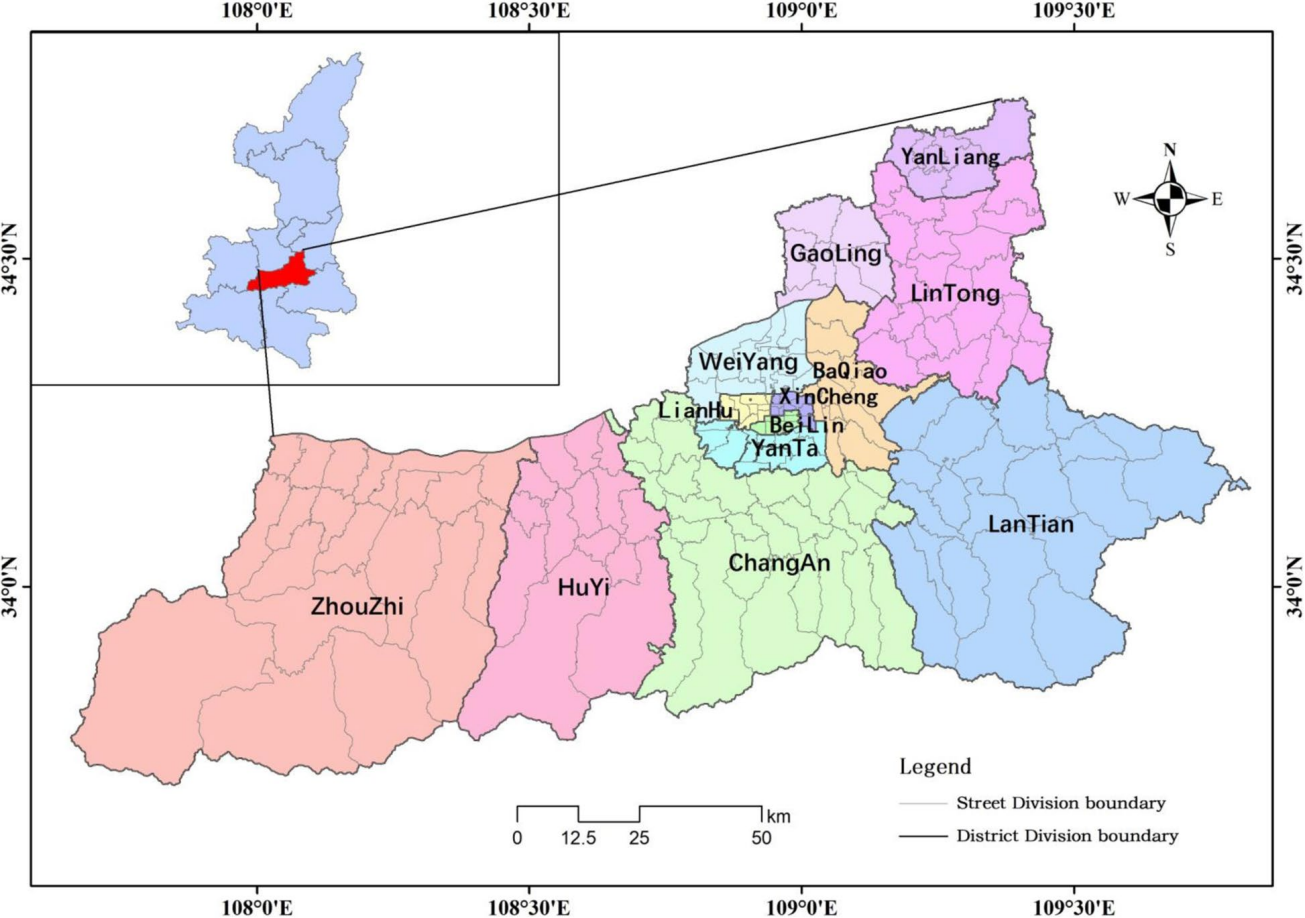
Administrative division of Xi’an and location in Shaanxi Province

Xi ‘an, the capital of Shaanxi Province, is located in the heart of the Guanzhong Plain in western China. It is also a national central city and a sub-provincial city. Xi ‘an consists of 11 districts and 2 counties, including 172 streets (towns), with a total area of 10,752 km^2^. In 2019, the number of permanent residents reached 10.2035 million (Fig. 1). There are 365 hospitals at all levels in the city, with a relatively complete emergency system, including an emergency center and 53 emergency stations. According to data from emergency services, there are fewer than five ambulances per 50,000 people. From the perspective of spatial distribution, 60% of medical facilities in Xi ‘an are concentrated in the second ring, while only a small part is distributed around the main urban area and suburban counties. As an important part of emergency care, the emergency system undertakes the important role to ensure the life safety of the residents in Xi’an. The purpose of considering the synchronous analysis of urban and suburban areas is to provide residents an opportunity to enjoy equality.

### Data sources

#### Research scale and travel time data

There are 5857 residential areas in the study area. Land use data were from Resources and Environmental Sciences and Data Center of Chinese Academy of Sciences, with a spatial resolution of 100 m. The data was resampled to 500 m through ArcGIS 10.2. The road network data were from OSM platform (https://www.openstreetmap.org/). Due to reflecting the precise time of emergency, the real-time data is adopted to supersede the travel time cost, using the API service data from Baidu map open platform (http://lbsyun.baidu.com/index.php?title=webapi/directionlite-v1). Generally, ambulance or driving is taken for emergency transportation, so driving mode is called upon when navigation function is selected. The normal emergency service was selected to an example to show the results of modeling and researching about the emergency service in this paper. The non peak time period accounts for a large proportion in the whole day, and most of the emergency events were occurred to the general situation. In addition, the huge amount of data also needed to be considered., so only the data of 9:00-17:00 in off-peak hours were collected. The data of time from each residential areas to the emergency stations or hospitals with good weather conditions and non-holidays in September 2020 were crawled by using Python.

#### Emergency resource data

**Fig. 2:**
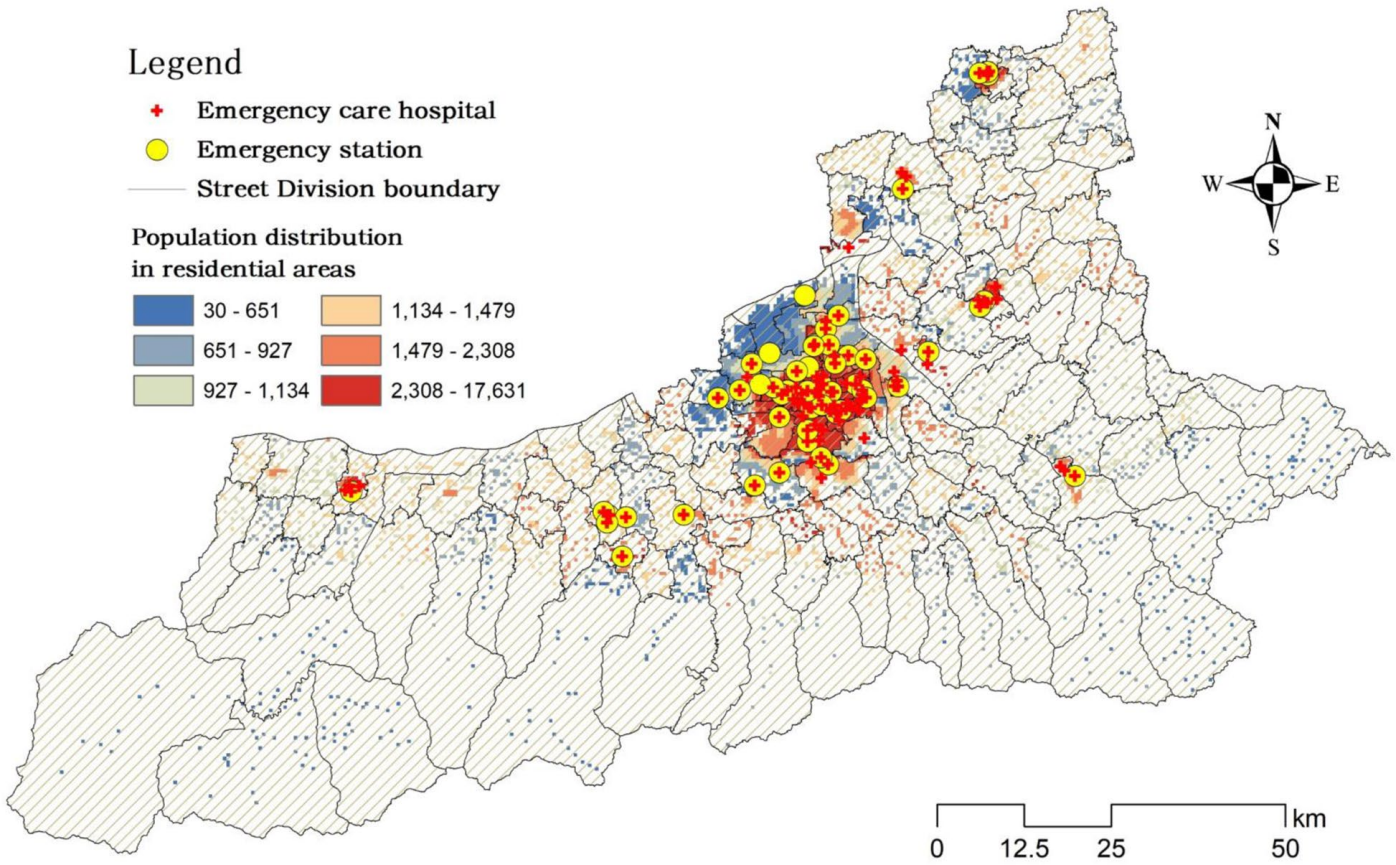
Spatial distribution of emergency care hospitals, emergency stations and population of residential areas in Xi’an

Medical resource data related to emergency care are from the official websites of Xi ‘an Health Commission, Emergency Center and hospitals. The names, locations and number of ambulances of the 53 emergency stations can be obtained from data. Moreover, the names, locations and corresponding number of beds provided by 104 hospitals with emergency capacity can also be obtained (Fig. 2).

#### Population data of residential areas

Population data were obtained from the Xi’an Bureau of Statistics, the Sixth National Population Census, and the Chinese Academy of Resources and Environmental Sciences and Data Center. Research units based on high spatial resolution can better reflect the differences in residents’ access to these resources. However, due to the lack of the latest street population data, we used the street population ratio of the sixth census data to allocate the total resident population and get the resident population of each street. Then the street population is allocated to each residential area according to the population density map provided, and the population of each residential area is finally obtained (Fig. 2).

## Methodology

### Improved potential model

Potential model is based on the law of universal gravitation to study the gravity value of regional social and economic development. Hansen first applied it in the evaluation of accessibility to measure the spatial distribution of activities at a certain location, or the target value that people expect to overcome certain resistance to get [31]. The gravity value here is denoted by ${A}_i^H$. The basic expression is as follows:

1 $${A}_i^H=\sum_{j=1}^n{A}_{ij}=\sum_{j=1}^n{S}_j{d}_{ij}^{-\beta }$$

where *A*_*ij*_ is the gravity generated by location j to location i, *S*_*j*_ is the service capacity or quality of the facility, *d*_*ij*_ is the travel distance or time from residential area settlement i to facility j, *β* is the travel friction coefficient, and n is the number of all facilities. It can be concluded that the closer the residential area is to the facility, the smaller the distance or time spent when the friction coefficient is fixed, and the more attractive the facility is to the population of the residential area.

The service capacity of the supply side is considered when analyzing resource allocation above equation, but competition within the demand side is not taken into account. Therefore, Weibull used the population scale to reflect the internal competition on the demand side in the improved model [32]. Joseph and Bantock was the first to apply this model to the accessibility evaluation of medical resources [33]. The improved model is expressed as follows:

2 $${A}_i=\sum_{j=1}^n\frac{S_J{d}_{ij}^{-\beta }}{V_j}$$

3 $${V}_j=\sum_{k=1}^m{P}_k{d}_{kj}^{-\beta }$$

where *A*_*i*_ is the accessibility value, n and m are the number of supply and demand respectively, *V*_*j*_ is the quantity of demand that may be received at the supply j, *P*_*k*_ is the population of location k, *d*_*kj*_ is the cost (time or distance) from the demand to the supply, and *β* is the travel friction coefficient.

**Fig. 3:**
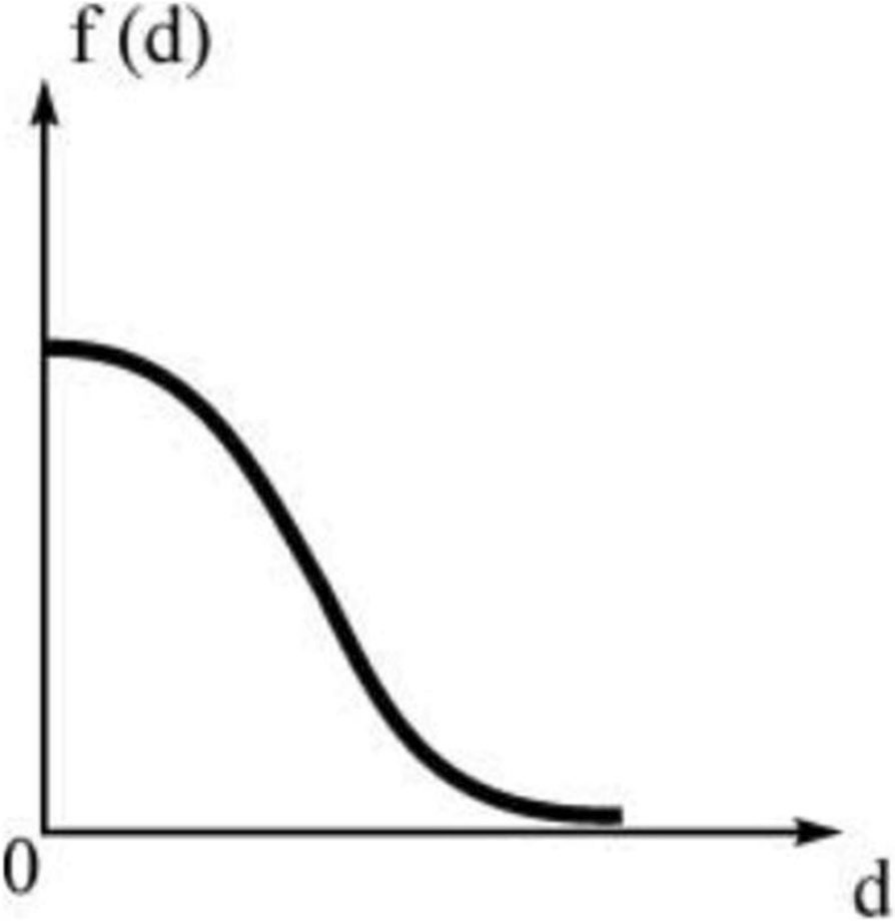
Conceptualizing distance decay with Gauss function

In fact, concerning the emergency services in the whole city, it is possible for any emergency station to send an ambulance to every residential area or even any place as long as there is a need. Therefore, it is not necessary to limit the service scope of the emergency station when evaluating the allocation of ambulance resources, but it can improve the service ability of the nearby station through the attenuation function, as well as reduce the service ability of the remote station. Referring to several studies on attenuation effect [34–37], *β* is replaced by Gauss function *f*(*d*) in this research (Fig. 3). In this way, it can be indicated that the ambulance service ability near is stronger, while the service ability far away is close to zero.

### The network map API service

The travel time of this paper is obtained through the path planning interface in the network map API service, which is implemented based on Python scripting language. Since the data calculated the travel time between the starting and ending points in the study area, the real time of emergency treatment could be taken into account in terms of real-time road conditions [23]. Firstly, the location coordinates of the residential area and the hospital are extracted as the starting point and the ending point respectively. They were imported into ArcGIS to generate OD matrix, and the corresponding relationship between all residential areas and each hospital was obtained respectively. Secondly, the geographic coordinates of each point are converted to map coordinates to match the API interface data. The real-time shortest time data from all residential areas to each hospital is obtained by calling the function.

### Inverted two-step floating catchment area method(i2SFCA)

The availability and proximity of service providers in measuring spatial accessibility are considered in the two-Step Floating Catchment Area Method(2SFCA). The ratio of supply and demand can reflect the convenience of obtaining services in residential areas, and it is usually used to evaluate the service of medical facilities [38]. The demand side oriented is focused on, the threshold range of facility services is determined by dichotomy, and the distance attenuation function is used to set the weight of the available services in this method [39, 40]. The expression of the model is as follows:

4 $${A}_i=\sum_{j=1}^n\left[{S}_jf\left({d}_{ij}\right)/\left(\sum_{k=1}^m{D}_kf\left({d}_{kj}\right)\right)\right]$$

Where *A*_*i*_ is the accessibility value, n and m are the number of supply and demand points respectively, *S*_*j*_ is the supply capacity, *P*_*k*_ is the demand in location k, *d*_*ij*_ and *d*_*kj*_ is the cost (time or distance) from demand to supply, and *f*(*d*_*ij*_) and *f*(*d*_*kj*_) represent the weight obtained by using the distance attenuation function.

Similar to the above model, Wang proposed the inverted 2SFCA (i2SFCA) to measure the “crowdedness” of service facilities after a series of theoretical derivation and case verification by transforming the supply side and demand side [41, 42]. This model represents the level of stress that the facility may experience due to the number of residents in the service area. This formula can be written as:

5 $${C}_j=\sum_{i=1}^m\left[{D}_if\left({d}_{ij}\right)/\left(\sum_{l=1}^n{S}_lf\left({d}_{il}\right)\right)\right]$$

Where *C*_*j*_ is interpreted as “projected or potential crowdedness” (e.g., patients per bed in a hospital, students per teacher in a school, clients per clerk, etc.). A higher *C*_*j*_ value indicates a service facility being more crowded (saturated, stressed, or busy). *f*(*d*_*ij*_) is a generalized distance attenuation function. Other variables can be referred to the Eq. (4). *f*(*d*_*ij*_) can be further expressed as follows:

6 $$f\left({d}_{ij}\right)=\left\{\begin{array}{c}g\left({d}_{ij}\right),\kern1em {d}_{ij}\le {d}_0\\ {}\kern2.5em 0,\kern1em {d}_{ij}>{d}_0\end{array}\right.$$

Where *d*_*ij*_ is the time distance between i and j, *d*_0_ is the search radius, which is the effective service radius of the facility, *g*(*d*_*ij*_) represents the distance decay function within the search radius. This is a segmented function. Within the search radius, the piecewise formula and the distance attenuation function of gravity model are used to distinguish the weights according to the distance, while the value outside the search radius is represented by constant zero.

In this paper, the service range of the hospital is set according to its service capacity, and the Gaussian attenuation mode is set for all the distances to the hospital. The service radius of the secondary hospital is set to 30 min, then the place beyond this time will not be provided service. Because the level of the tertiary hospital is high, they can serve any location in the whole city without threshold.

### Three stages of emergency and processing

**Fig. 4:**
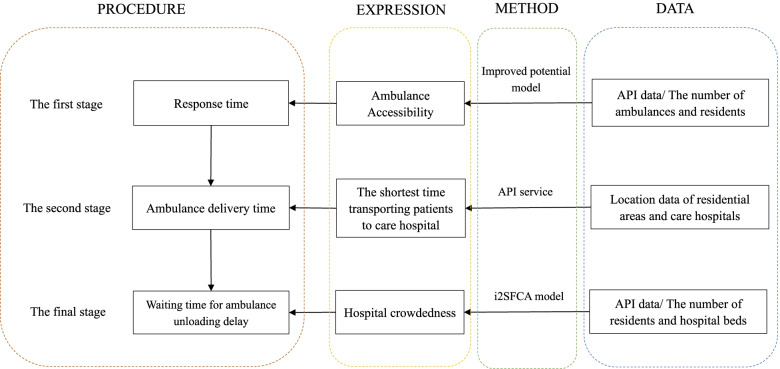
Three stages procedure of EMS

The time from the occurrence of an emergency to gaining medical assistance is called the inherent time of emergency system, which mainly includes the time of dispatching the ambulance, the time of transporting the patient to the hospital, and the time when the ambulance may face the delay of unloading the patient. Different indexes were obtained by different calculation methods to represent the time required to obtain the corresponding emergency resources (Fig. 4).

The first stage is from the beginning of the incident to the deployment of ambulances to the scene of the incident. During this process, the number of ambulances around and the time of arrival at the scene of the incident need to be considered. The response time generated in this phase is measured by the ratio of supply and demand of ambulances and residents in the area, which is calculated by an improved gravity model. The calculation results can reflect how convenient it is for people to obtain the ambulance. Higher values indicate easier access to resources and a shorter response time for ambulances.

In the second stage, the patient is transported to the nearest hospital that can provide emergency medical services for further treatment after obtaining an ambulance. The time cost is calculated by obtaining the shortest time distance from the path planning data which can reflect the real time condition. The less time spent in this process, the better. To a certain extent, it also reflects the impact of the geographical distribution of emergency care hospitals on the delivery time.

The final stage is the unloading process after the patient arrives at the hospital. Due to the lack of hospital beds, patients may not be unloaded on arrival timely resulting in increased waiting times. This crowdedness is measured by the ratio of residents to inpatient beds with i2SFCA, and assign the crowdedness index of the nearest hospital to the residential area. Higher crowdedness index means longer waiting times and more difficulty in obtaining resources.

## Results

### Response time

**Fig. 5:**
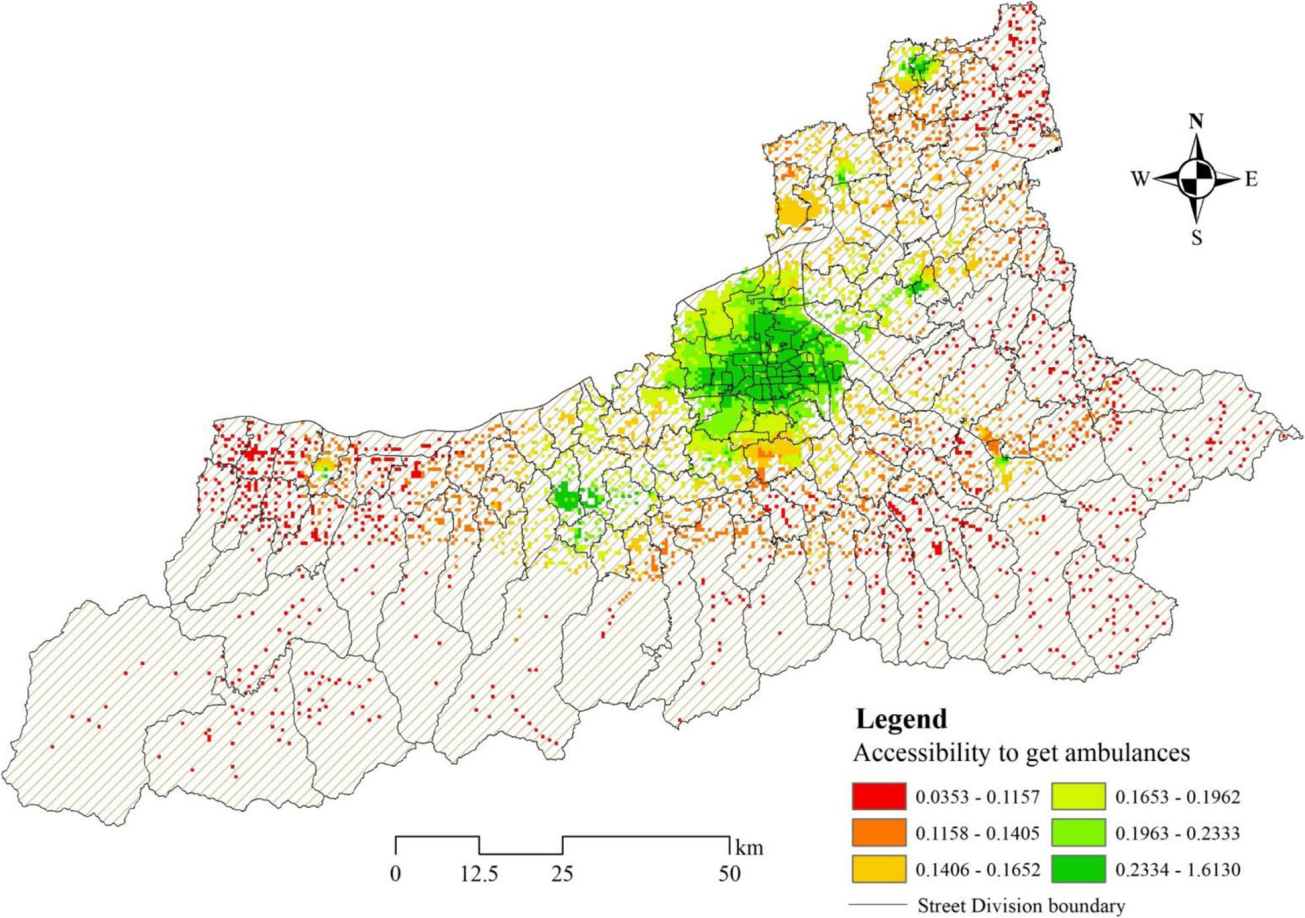
Spatial distribution in residential areas of accessibility to get ambulances (response time)

When analyzing the response time of the ambulance, we considered the supply of the ambulance and the time it takes for the ambulance to arrive at the incident. The calculated result is characterized as an accessibility value and visualized (Fig. 5). The figure shows that there are differences between residents in urban built-up areas and suburban areas in accessing to ambulance resources. In urban built-up areas emergency stations are more numerous and densely distributed, making it easier for residents to obtain ambulance resources than those in suburban areas, while the accessibility distribution within the whole built-up area is uneven. We can see that residents in the inner of city have better access to ambulances than those in the north and south city, but there are also points in the inner city where accessibility is lower. These less accessible areas are under greater competition and pressure for access to ambulance resources due to the large population or the poor traffic capacity of roads in the same conditions. Furthermore, in the suburban areas, it can be seen that the convenience of obtaining ambulances in residential areas is distributed in a circle structure. The number of ambulances is relatively sufficient in the core area of the suburban areas, while the areas outside the core areas are far away from the emergency stations, making it very difficult for residents to access the ambulance resources. These places are also vulnerable areas of the whole city’s emergency system services.

According to the statistics of the ability to obtain ambulance resources in Xi’an residential areas in 2 counties and 11 districts of Xi’an city (Table 1), the mean in the whole city of ambulance response time accessibility is 0.1717, and the standard deviation is 0.0642. The areas where the mean is lower than 0.2 are the suburban areas of Xi’an, and the mean value of built-up areas is higher than the overall level of the whole city. In terms of intra-regional differences, only Huyi District has the highest standard deviation of more than 0.1, while the standard deviation of the rest 12 districts is lower than the mean of the whole city. There are obvious differences in the access to ambulance resources among the residential areas in Huyi districts, and there are serious polarization. Otherwise, we found that more than 95% of the population, respectively in Lantian County and Zhouzhi County, have lower access to ambulance resources than the city’s average level. Some residential areas in the built-up area may not have timely access to resources. Although the overall level of Weiyang District and Yanta District is relatively high, there are still respectively 3.27 and 4.69% of the residents whose ability to obtain ambulance resources is lower than the average level of the whole city. The poor areas to access inside the city still need attention, especially at the edge of the urban built-up area. More medical resources should be allocated in these areas to narrow the gap with the center of the built-up areas.

**Table 1:** Data statistics of accessibility to get ambulances in different districts of Xi’an

District	Mean	Standard deviation	Below the district mean			Below mean in the whole city		
			streets (%)	residential areas (%)	population (%)	streets (%)	residential areas (%)	population (%)
Baqiao	0.2048	0.0401	55.55	49.59	42.82	22.22	23.85	21.25
Beilin	0.2701	0.0393	37.50	66.67	66.77	0.00	0.00	0.00
Chang’an	0.1537	0.0324	60.00	50.89	54.60	80.00	70.16	74.78
Gaoling	0.1582	0.0573	71.43	71.66	65.55	85.71	89.17	85.79
Huyi	0.1878	0.1005	71.43	68.13	59.66	64.29	52.39	45.03
Lianhu	0.2643	0.0456	66.67	59.17	54.90	0.00	0.00	0.00
Lintong	0.1469	0.0379	65.22	60.97	56.59	86.96	80.48	77.80
Weiyang	0.2158	0.0471	58.33	59.72	40.30	0.00	5.52	3.27
Xincheng	0.2555	0.0324	44.44	45.53	37.59	0.00	0.00	0.00
Yanliang	0.1548	0.0551	57.14	58.94	48.07	57.14	73.17	60.44
Yanta	0.2063	0.0275	40.00	52.11	50.99	0.00	9.05	4.69
Lantian	0.1171	0.0297	63.16	57.63	50.85	100.00	97.43	96.47
Zhouzhi	0.1055	0.0264	50.00	40.86	29.66	100.00	99.00	97.93

### Ambulance delivery time

**Fig. 6:**
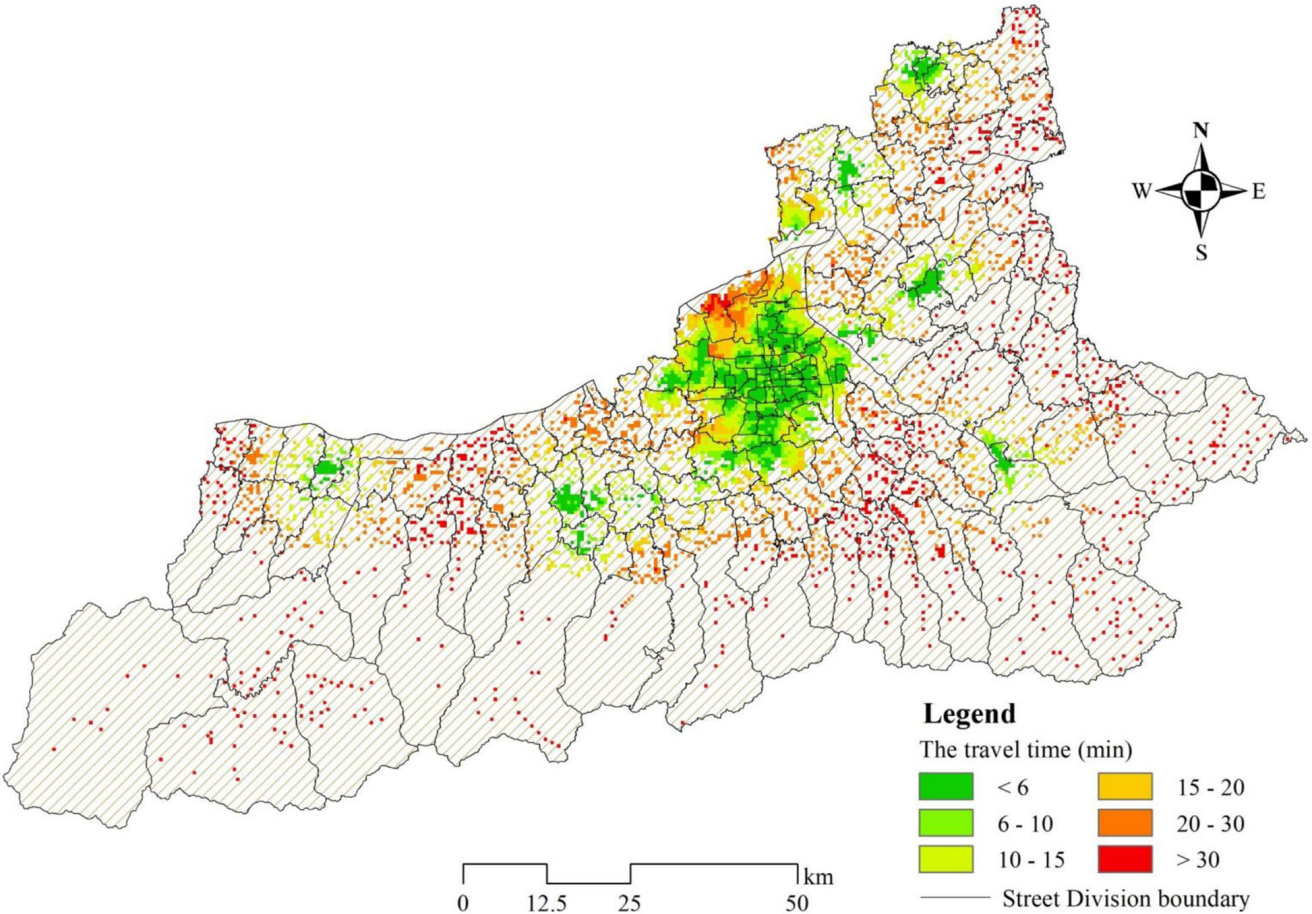
Spatial distribution of the shortest time in residential areas for an ambulance to transport patients to the nearest care hospital

The Fig. 6 shows the shortest time required for an ambulance to return to the care hospital from each residential area. The spatial distribution of the time from the residential areas to the care hospitals in the built-up area is cluster, which is closely related to the geographical location and distribution of the emergency hospitals. Due to the lack of hospitals in the north and southwest of the built-up area, the resistance in the process of returning may increase significantly, and it may take more time to reach the nearest hospital. The hospitals in the built-up area are densely distributed, but there are still some scattered residential areas that need to spend a long time in further obtaining hospital care. The accessibility to care hospitals is still poor in residential areas within the fringe of built-up areas. The residential areas located in core of suburb are close to hospitals, while the ones on the core periphery spend more time on obtaining hospital care as a result of the low density of road network and no hospital distribution. These areas are mainly distributed in the southern mountainous areas and the urban boundaries.

**Fig. 7:**
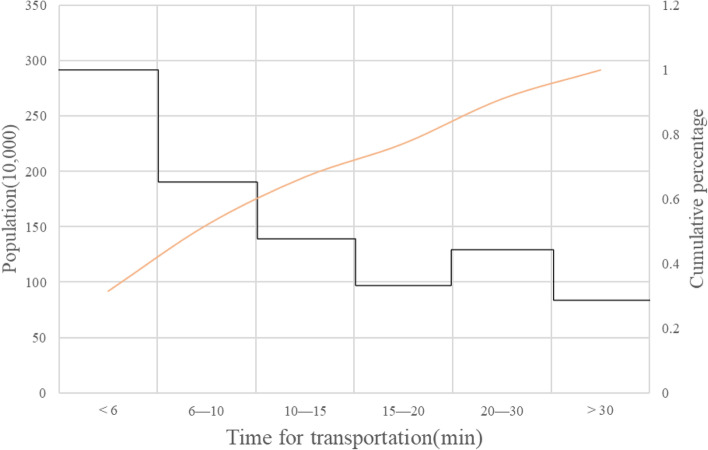
Statistics of the number of people arriving at emergency care hospital in different time

Figure 7 shows the number of people who can reach the nearest hospital within 6 min, 10 min, 15 min, 20 min, 30 min and more than 30 min respectively. 52% of the city’s population can reach the hospital within 10 min for further emergency care, and the number of residents who can reach the nearby hospital within 6 min for further care is the largest, reaching 2.9 million. However, there are still 9% of the residents, about 0.82 million people, who need to take more than 30 min to further obtain the care resources they need.

### Waiting time for ambulance unloading delay

**Fig. 8:**
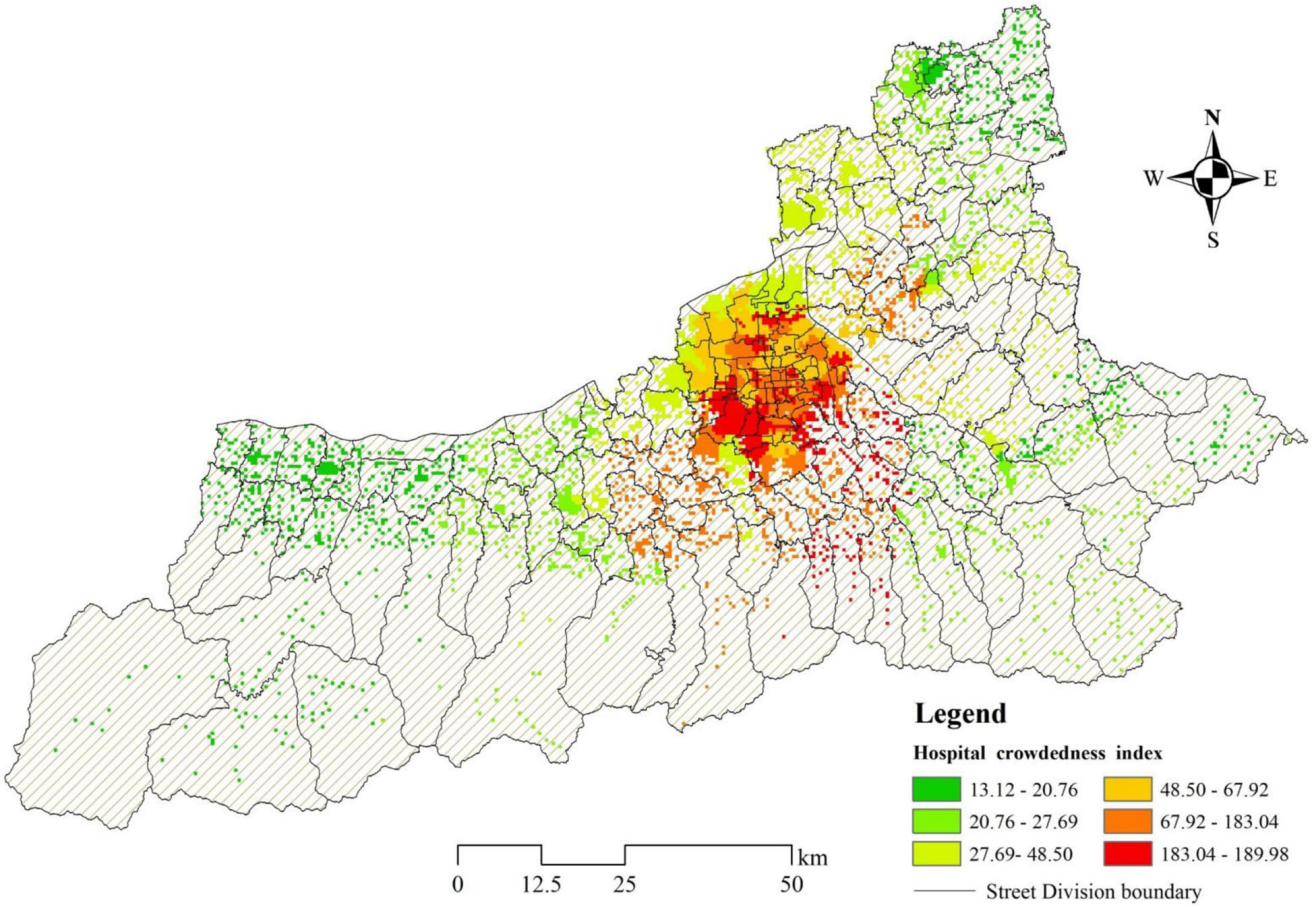
Spatial distribution of waiting time for different residential areas to arrive at care hospitals (hospital crowdedness index)

The crowdedness index of the corresponding nearest hospital was used to evaluate the possible waiting time of different residential areas for medical treatment. Larger value means longer waiting time (Fig. 8). The average crowdedness index of tertiary hospitals is 183, while that of secondary hospitals is only 50. It confirms the actual situation that the tertiary hospitals serve the population of the whole city, while the secondary hospitals serve the population nearby. The tertiary hospitals are more concentrated in the city center, which serve a larger population. For residents near these hospitals, the waiting time after arriving at the destination hospital may be longer, with up to 190 people competing for a bed at the same time. Especially in the southwest area of the built-up area, there are more tertiary hospitals and fewer secondary hospitals, so the residential areas in this area may face great crowdedness when choosing medical treatment nearby. In addition, due to the lack of secondary care hospitals in the southeast, the suburban residential areas are also identified as high crowdedness as a result of closer to tertiary care hospitals. In contrast, hospitals in the suburban areas are mainly secondary hospitals, serving a smaller population than those in the built-up area. Therefore, in the case of the same medical level, the secondary hospitals in the built-up area have a higher index of crowdedness, around which the residents have a longer waiting time when they are looking for hospital beds.

### Identify the areas with insufficient access to emergency medical resources

**Fig. 9:**
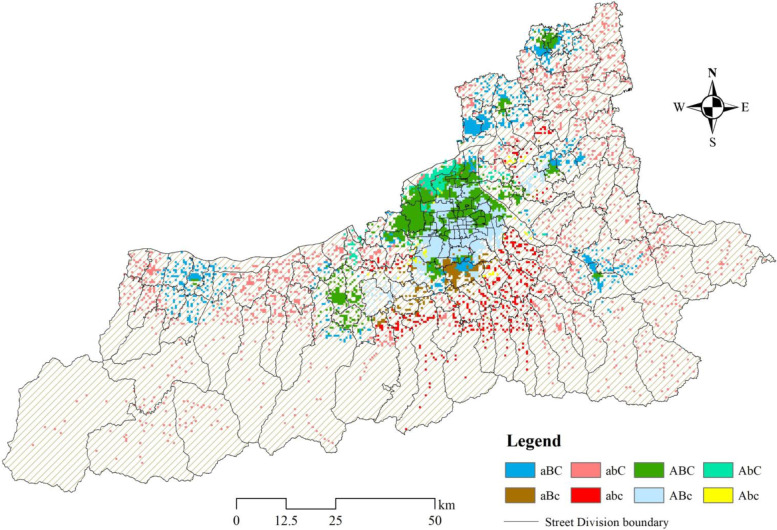
Spatial distribution of different combinations by the comprehensive analysis on the three stages of emergency in residential areas

We used some combinations of letters to identify the level of accessibility of different areas. A means greater than the mean of the city’s response time accessibility, while a means less than the mean. B means less than the mean of the shortest time accessibility, while b means greater than the mean. And C means less than the mean of crowdedness index, while c means greater than the mean. Based on the results of the above three stages of emergency, they are grouped into eight categories. Different combinations can characterize the differences in the convenience of getting emergency resources in residential areas. At the same time, some vulnerable regions with low accessibility to emergency care are also identified (Fig. 9, Table 2). abc is expressed as the most vulnerable area of emergency medical services in the whole city. The residents with a number of 495,500 have the worst convenience in obtaining emergency resources there, which mainly distributed in the suburban areas of the south of the urban built-up area. At present, there is urgent need to strengthen the distribution of emergency resources in these high-risk areas to reduce the risk of life threats to residents (red area in the Fig. 8). aBc、abC、Abc indicate that residents have poor convenience in obtaining two of the resources in the three stages of emergency, which is called the intermediate risk regions including a total of 21.92% of the population. abC has the largest population among the three combinations mentioned, reaching 1.62 million. These residential areas are mainly distributed in the southern mountainous areas and urban fringe, which are not well served by existing ambulance and hospital resources. In addition, 46.11% of the population is in the low- risk regions of emergency services, mainly due to the long time spent in obtaining one of the resources. The population of ABc of about 3 million is the largest proportion of the population in all combinations, signifying the most residents need to consider the problem of hospital crowdedness. Therefore, in the layout of emergency medical resources, we should not only consider the distribution and quantity in the outer suburban areas, but also consider the balance between the expansion and utilization of medical resources in the central area of the city.

**Table 2:** Statistics on the number and percentage of residential areas and population in different combinations

Combination	Residential areas		Population	
	Number	Percentage (%)	Number (10,000)	Percentage (%)
aBC	908	15.5	106.88	11.6
aBc	238	4.06	33.32	3.62
abC	1637	27.95	161.76	17.55
abc	396	6.76	49.55	5.38
ABC	1339	22.86	245.03	26.59
ABc	1048	17.89	300.39	32.6
AbC	240	4.1	17.63	1.91
Abc	50	0.85	6.89	0.75

## Discussion

This research discusses the emergency process in different time periods, and measures the response time, delivery time and waiting time through suitable indicators for each stage. The main purpose is to indicate the impact of getting ambulances and hospital beds and road condition on the emergency time, which can provide more detailed reference for improving the quality of emergency service system and achieve targeted.

The response time of ambulance is a key to evaluate the accessibility of emergency resources in an area. Some scholars use descriptive statistics or historical records to evaluate the parameter [43], which do not consider the competition. Because ambulance is a kind of public resource, which is likely to be occupied by others when you need it. The gravity model was adopted to consider the time for the ambulance to go to the incident, and competition with surrounding residents in this paper. Based on the previous historical records, some studies believe that the ambulances provided by the nearest three emergency stations can basically meet the scheduling requirements. Therefore, the threshold of the service capacity of the emergency station to each residential area is determined at the three nearest one when calculating accessibility [44].But this method is more suitable for urban built-up areas where emergency resources are concentrated. For the suburban areas, the service capacity of the emergency station will be increased artificially because of generally the set of emergency stations of no more than three, so the accuracy of the assessment needs to be considered further. In this study, we assume that every emergency stations can serve all residents of the city if needed, but the possibility of remote emergency stations service is reduced by using the Gaussian attenuation model. It is consistent with the theoretical situation that ambulances can be dispatched from any emergency station whenever there is a need, also with the actual situation that several emergency stations nearby can basically meet the demand.

After the patients have obtained the ambulance service, they need to be transported to the nearby hospital which can provide further medical care. Considering it is crucial for patients to arrive at the hospital earlier, the time ambulances return to the nearest hospital by the API data which can reflect the real road conditions is evaluated. This is conformed with common sense and the habits of the vast majority of people. As for the wishes of the patient and his family [45], however, the ambulance may not return to the nearest hospital, thus making the location of the return uncertain. This case is difficult to make systematic analysis and evaluation because of uncertainty. Through the study of time distance, we find that road construction plays an important role in improving accessibility. The northern district of the built-up area of Xi ‘an is a new urban expansion area. Due to the imperfect road facilities, it still takes a long time to drive to the city center. In addition, because of the expressways in some suburban areas, the time spent by residents who live in which close to the entrances and exits of expressways to built-up areas is obviously less than that spent by other residents. In order to reduce the traffic time between residential areas and emergency stations, it is necessary to speed up the construction of urban expressway and ring highway so that the advantageous resources of the city center can be obtained at a faster speed. At last, through the data processing of the average travel time of multi period in the off-peak, it is found that some roads still have poor traffic ability and congestion, which also increases the requirements for ambulance transportation. It needs to be further considered that road congestion may affect the emergency time in the peak period [46, 47].

Overcrowding in hospitals is a major cause of delay in ambulance unloading, and the waiting time can be calculated by the corresponding crowding value through the i2SFCA method [11, 12, 41, 42]. When evaluating the crowdedness corresponding to residential areas, we considered returning to the nearest hospital based on the principle of proximity in the previous stage, and assigned the crowdedness of the hospital to the residential area. It’s not a possibility to determine the overall crowding environment of hospitals to residential areas, and we cannot well take into account factors such as human choice and force majeure, which may be an issue that can be further studied in the future. On the other hand, there is a lot of competition for beds in some of the tertiary hospitals with high reputation and medical level. Some residential areas have been identified with overcrowding conditions, especially the areas surrounded by tertiary hospitals in built-up area. It is true that these areas are close to the tertiary hospitals, but it is also likely that the waiting time is longer due to the overcrowding of the hospital, and the results are not satisfactory in the overall evaluation. Therefore, one can consider the nearby secondary hospital according to his condition to reduce the pressure on the tertiary hospital and increase the utilization rate of the secondary hospital, while this requires a good Hierarchical diagnosis and treatment system [48, 49]. In addition, secondary hospitals and tertiary hospitals should be reasonably staggered in space. If there are only tertiary hospitals or secondary hospitals in a certain range, it may cause the problem of crowding or referral delay. According to the results, the low level of crowding in secondary hospitals does not mean that residents have high accessibility to medical resources. Because the data shows that even in the hospitals with the lowest competitive pressure, there are still 14 people competing for one bed. At the same time, the impedance caused by time distance is also a significant factor. Finally, some articles indicate that the quickest way to evaluate the emergency medical resources of a place is to stay in the local emergency room for a few hours [50]. It may be more accurate and time to use the survey data to judge the crowdedness and the computer system to detect the hospital crowdedness [51–53], but it is a new attempt to use the i2SFCA method to assess.

The limitation of the research is this study measured the supply-demand ratio of access to resources rather than the actual time spent in emergency treatment it is indeed more meaningful if the real time can be obtained. Furthermore, The service scope of emergency station and emergency hospital is not clear, since there is no unifying method for time selection yet for emergency accessibility, future research could explore a systematic way for selecting time based on various parameters such as locations characteristics, nature of the emergency, and type of responders [17].

The main selection bias in our study is that all groups are considered, but it is clear that vulnerable groups such as the elderly and pregnant women are in greater need of emergency services. In addition, there is no standard to measure the accessibility of emergency services, so the results of the three stages can not be unified, which leads to information bias. Later, the distribution of different groups can be considered and a new approach to accessibility that can unify the results of the three phases will be explored in the future to simulate real emergency processes for better evaluation.

There are many factors that may affect the evaluation results, such as infrastructure construction and management, actual service demand, system equipment, preferences of different groups and traffic congestion [45], and even precipitation and natural disasters [54]. The accessibility results are different due to different factors in different periods. For example, during the COVID-19 outbreak in Xi’an, the whole city had been locked down. At that time, the roads were unobstructed, and people’s demand for emergency medical resources was different from that in ordinary periods, resulting in dissimilarity results. On the other hand, we all know, urban traffic congestion has a large significant impact on accessibility in the peak period, which will also produce some different results [47]. Therefore, the analysis of the accessibility of emergency medical services under different scenarios based on the new evaluation method proposed in this paper may be beneficial to further research.

## Conclusions

In this paper, based on the map API service data, we used the improved potential model, and i2SFCA method to evaluate the possible time required to obtain the emergency medical services. The evaluation results can reflect the difference in obtaining emergency medical resources accessibility in different residential areas, and identify the vulnerable regions of the emergency service in the city. The results show that residents living in the built-up area where emergency resources are concentrated spend less time on response time and delivery time, and it is more convenient to obtain ambulance resources and arrive at care hospitals, while the most prominent problem is that they may face serious hospital crowdedness. The suburban residents may face low hospital crowdedness, but they spend more time on getting ambulances and going to care hospitals. So the first priority in the suburban areas is to strengthen the layout of a certain number of emergency stations and care hospitals to make up for the lack of supply. Through the identification of residential areas facing emergency service risk, the high-risk areas for access to emergency medical services are mainly distributed in suburban residential areas in the south of the city center, involving about 0.50 million residents. There are 2.02 million residents at medium risk areas, where mainly in the southern mountainous areas and the periphery of the core suburban areas of the city. Another 4.25 million residents are in low-risk areas, which are mainly distributed in built-up areas. For residents in the central area of the built-up area, the problem of hospital crowdedness needs to be solved. For residents around the center of built-up areas, the problem of allocation of ambulance resources needs to be solved. And for residents in the suburban areas, there is a dual problem of ambulances and care hospitals allocation to be solved. How to make up for the lack of facilities in each region in the future can refer to our evaluation results, which is conducive to the local special measures to improve their own deficiencies.

## Data Availability

The datasets used and/or analysed during the current study are available from the corresponding author on reasonable request.

## Abbreviations

EMS: Emergency medical service
OSM: Open street map
2SFCA: Two-step floating catchment area
API: Application programming interface
i2SFCA: Inverted two-step floating catchment area
